# Adenomas from individuals with pathogenic biallelic variants in the *MUTYH* and *NTHL1* genes demonstrate base excision repair tumour mutational signature profiles similar to colorectal cancers, expanding potential diagnostic and variant classification applications

**DOI:** 10.1016/j.tranon.2024.102266

**Published:** 2025-01-09

**Authors:** Romy Walker, Jihoon E. Joo, Khalid Mahmood, Mark Clendenning, Julia Como, Susan G. Preston, Sharelle Joseland, Bernard J. Pope, Ana B.D. Medeiros, Brenely V. Murillo, Nicholas Pachter, Kevin Sweet, Allan D. Spigelman, Alexandra Groves, Margaret Gleeson, Krzysztof Bernatowicz, Nicola Poplawski, Lesley Andrews, Emma Healey, Steven Gallinger, Robert C. Grant, Aung K. Win, John L. Hopper, Mark A. Jenkins, Giovana T. Torrezan, Christophe Rosty, Finlay A. Macrae, Ingrid M. Winship, Daniel D. Buchanan, Peter Georgeson

**Affiliations:** a Colorectal Oncogenomics Group, Department of Clinical Pathology, The University of Melbourne, Parkville, VIC, 3010, Australia; b University of Melbourne Centre for Cancer Research, Victorian Comprehensive Cancer Centre, Parkville, VIC, 3010, Australia; c Melbourne Bioinformatics, The University of Melbourne, Melbourne, VIC, 3053, Australia; d Clinical and Functional Genomics Group, International Research Centre/CIPE, A.C. Camargo Cancer Centre, Sao Paulo, 01508-010, Brazil; e Genetic Services of Western Australia, King Edward Memorial Hospital, Perth, WA, 6008, Australia; f Medical School, University of Western Australia, Perth, WA, 6009, Australia; g School of Medicine, Curtin University, Perth, WA, 6845, Australia; h Division of Human Genetics, Department of Internal Medicine, College of Medicine, The Ohio State University, Columbus, OH, 43210, USA; i Hunter Family Cancer Service, Newcastle, NSW, 2298, Australia; j St Vincent's Cancer Genetics Unit, Sydney, NSW, 2290, Australia; k Surgical Professorial Unit, UNSW Clinical School of Clinical Medicine, Sydney, NSW, 2052, Australia; l Adult Genetics Unit, Royal Adelaide Hospital, Adelaide, SA 5000, Australia; m Adelaide Medical School, University of Adelaide, Adelaide, South Australia, Australia; n Hereditary Cancer Centre, Prince of Wales Hospital, Randwick, New South Wales, Australia; o School of Medicine and Public Health, University of Newcastle, Callaghan, New South Wales, Australia; p Prince of Wales Clinical School, Faculty of Medicine, University of New South Wales, Randwick, New South Wales 2031 Australia; q Illawarra Cancer Care Centre, Wollongong Hospital, Wollongong, New South Wales 2500 Australia; r Lunenfeld Tanenbaum Research Institute, Mount Sinai Hospital, University of Toronto, Toronto, ON, Canada; s Division of Medical Oncology and Hematology, Princess Margaret Cancer Centre, University Health Network, Toronto, ON, Canada; t Centre for Epidemiology and Biostatistics, Melbourne School of Population and Global Health, VIC, 3053, Australia; u National Institute of Science and Technology in Oncogenomics and Therapeutic Innovation, Sao Paulo 01508-010, Brazil; v Envoi Specialist Pathologists, Brisbane, QLD, 4059, Australia; w University of Queensland, Brisbane, QLD, 4072, Australia; x Genomic Medicine and Family Cancer Clinic, Royal Melbourne Hospital, Parkville, VIC, 3000, Australia; y Department of Medicine, The University of Melbourne, Parkville, VIC, 3000, Australia; z Colorectal Medicine and Genetics, The Royal Melbourne Hospital, Parkville, VIC, 3000, Australia

**Keywords:** Mutational signature, Adenoma, SBS36, SBS30, MUTYH, NTHL1, Variant of uncertain clinical significance

## Abstract

**Background:**

Colorectal cancers (CRCs) from people with biallelic germline likely pathogenic/pathogenic variants in *MUTYH* or *NTHL1* exhibit specific single base substitution (SBS) mutational signatures, namely combined SBS18 and SBS36 (SBS18+SBS36), and SBS30, respectively. The aim was to determine if adenomas from biallelic cases demonstrated these mutational signatures at diagnostic levels.

**Methods:**

Whole-exome sequencing of FFPE tissue and matched blood-derived DNA was performed on 9 adenomas and 15 CRCs from 13 biallelic *MUTYH* cases, on 7 adenomas and 2 CRCs from 5 biallelic *NTHL1* cases and on 27 adenomas and 26 CRCs from 46 non-hereditary (sporadic) participants. All samples were assessed for COSMIC v3.2 SBS mutational signatures.

**Results:**

In biallelic *MUTYH* cases, SBS18+SBS36 signature proportions in adenomas (mean±standard deviation, 65.6 %±29.6 %) were not significantly different to those observed in CRCs (76.2 % ± 20.5 %, *p-value*=0.37), but were significantly higher compared with non-hereditary adenomas (7.6 % ± 7.0 %, *p-value*=3.4 × 10^–4^). Similarly, in biallelic *NTHL1* cases, SBS30 signature proportions in adenomas (74.5 %±9.4 %) were similar to those in CRCs (78.8 % ± 2.4 %) but significantly higher compared with non-hereditary adenomas (2.8 % ± 3.6 %, *p-value*=5.1 × 10^–7^). Additionally, a compound heterozygote with the c.1187G>*A* p.(Gly396Asp) pathogenic variant and the c.533G>C p.(Gly178Ala) variant of unknown significance (VUS) in *MUTYH* demonstrated high levels of SBS18+SBS36 in four adenomas and one CRC, providing evidence for reclassification of the VUS to pathogenic.

**Conclusions:**

SBS18+SBS36 and SBS30 were enriched in adenomas at comparable proportions to those observed in CRCs from biallelic *MUTYH* and biallelic *NTHL1* cases, respectively. Therefore, testing adenomas may improve the identification of biallelic cases and facilitate variant classification, ultimately enabling opportunities for CRC prevention.

## Highlights

• Adenoma-derived mutational signatures can identify inherited cancer syndromes.
• Adenoma-derived mutational signatures can aid germline variant classification.
• Factors that influenced signatures included MMR-deficiency and serrated polyp type.
• *KRAS* c.34G>*T* is a more accurate biomarker of biallelic *MUTYH* in CRCs than adenomas.

## Graphical abstract

**Figure:**
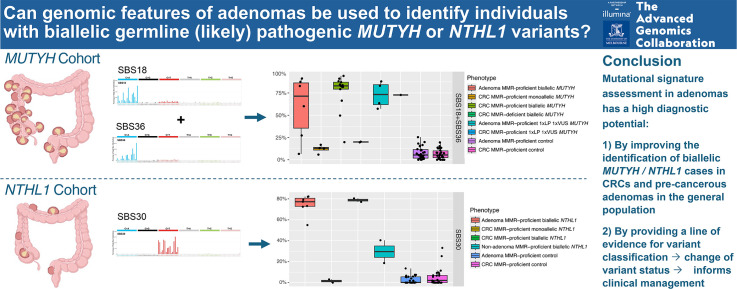

## Introduction

Identifying people who have an increased risk of developing colorectal cancer (CRC), including people with a hereditary CRC or polyposis syndrome, provides important opportunities for cancer prevention. Individuals with homozygous or compound heterozygous likely pathogenic or pathogenic (LP/P) variants in the base excision repair genes *MUTYH* [1] and *NTHL1* [2] (i.e., biallelic cases) predispose to the development of multiple pre-cancerous adenomas in the colon (adenomatous polyposis), CRC and a spectrum of extra-colonic cancers [2,3].

The application of tumour mutational signature profiling to identify hereditary cancer syndromes related to DNA repair defects has been highlighted [4]. Single base substitution (SBS) and insertion/deletion mutational signatures in CRC have been shown to be accurate predictors of Lynch syndrome and biallelic germline LP/P variants in *MUTYH* [5]. In particular, the combination of SBS18 and SBS36 (SBS18+SBS36) can accurately identify those with germline biallelic *MUTYH* LP/P variants [5,6], while for *NTHL1*, the SBS30 mutational signature has been identified in CRCs from those with biallelic *NTHL1* LP/P variants [7]. Moreover, we previously identified two recurrent somatic mutations, namely the *KRAS* c.34G>*T* p.(Gly12Cys) and the *PIK3CA* c.1636C>A p.(Gln546Lys) mutations, that were strongly enriched in CRCs from biallelic *MUTYH* cases compared with CRCs from non-hereditary/sporadic cases (*KRAS: p-value*=1.4 × 10^–6^, *PIK3CA: p-value*=3.4 × 10^–4^) [6].

A further application of tumour mutational signature profiling is to aid variant classification. Previously, we have shown the presence of elevated levels of SBS18+SBS36 in CRCs provided evidence for an LP/P classification for the germline *MUTYH* variants c.1141G>*T* p.(Gly381Trp) and c.577–5A>G, where the second allele of *MUTYH* harboured an LP/P variant [6]. Alternatively, the absence of high levels of SBS18+SBS36 in CRCs supported a benign classification for *MUTYH* variants c.912C>G p.(Ser304Arg), c.821G>*A* p.(Arg274Gln), c.925C>T p.(Arg309Cys) and c.1431G>C p.(Thr477Thr) [6].

While these genomic features have been shown to be effective with CRC-derived data, there are important implications that could be facilitated by the ability to utilise mutational signature profiling in pre-cancerous adenomas namely: 1) identifying biallelic cases early before they develop cancer, including guiding surgical versus endoscopic management decision making, 2) enable pre-emptive genetic counselling and guide patient management strategies through risk assessment, 3) indicate if a second “unidentified” LP/P variant is present in monoallelic LP/P variant carriers, and 4) provide evidence for pathogenicity for variants of uncertain significance (VUS).

The aim of this study was to profile and compare the SBS18+SBS36 and SBS30 mutational signatures in adenomas and CRCs from biallelic *MUTYH* and biallelic *NTHL1* cases, respectively, with sporadic adenomas and CRCs from participants without a hereditary CRC/polyposis syndrome to determine their discriminatory potential and ability to inform variant classification.

## Material and methods

### Study cohort

Participants were men and women recruited to one of the following studies: 1) Applying Novel Genomic approaches to Early-onset and suspected Lynch Syndrome colorectal and endometrial cancers (ANGELS, *n* = 4), 2) Colorectal Cancer Family Registry (CCFR, *n* = 21) or 3) Genetics of Colonic Polyposis Study (GCPS, *n* = 5) who were identified to have either germline biallelic *MUTYH* or germline biallelic *NTHL1* LP/P variants from clinical diagnostic or research genetic testing. Formalin-fixed paraffin embedded (FFPE) tissue was collected for tumour mutational signature profiling comprising:

1) 9 adenomas and 15 CRCs from 13 biallelic *MUTYH* cases;
2) 4 CRCs from 4 monoallelic *MUTYH* cases;
3) 7 adenomas, 1 hyperplastic polyp, 1 traditional serrated adenoma and 2 CRCs from 7 biallelic *NTHL1* cases and
4) 2 CRCs from 2 monoallelic *NTHL1* cases.

A reference/control group of 46 participants from the CCFR who developed mismatch repair (MMR)-proficient adenomas (*n* = 27) and/or MMR-proficient CRCs (*n* = 26) and who were confirmed to not carry LP/P variants in 16 hereditary CRC/polyposis genes as defined in Seifert et al. [8] (i.e., non-hereditary/sporadic cases) were included in this study. The studies were approved by the respective ethics committees and institutional review boards. All participants provided written informed consent for collection of tissue and peripheral blood samples.

### Tissue and blood DNA extraction

Assessment of MMR-deficiency and extraction of DNA for participants from the CCFR was conducted as previously described [9]. For participants of the ANGELS and GCPS studies, the following protocol was implemented. First, an H&E-stained slide was assessed by a pathologist to identify areas of high tumour cellularity and adenoma content for macrodissection. FFPE tissue DNA was then extracted from the tumour and adenoma tissue using the QIAamp DNA FFPE Tissue Kit (Qiagen, Hilden, Germany). Blood DNA was extracted from peripheral lymphocytes using the DNeasy blood and tissue kit (Qiagen, Hilden, Germany) and sequenced as a matched germline reference.

### Whole-exome sequencing and bioinformatic analysis

Adenoma and CRC tissue DNA and matched blood-derived DNA underwent whole-exome sequencing (WES) using the SureSelect Clinical Research Exome v.2 kit (Agilent Technologies, Santa Clara, CA, United States), to a median depth of 357.9 reads (interquartile range (IQR)=287.8–464.0) for FFPE tissue DNA samples and median depth of 179.1 reads (IQR=118.1–204.6) for blood-derived DNA samples. Somatic single-nucleotide variants and short insertion/deletions were determined using the intersection of calls from Strelka (v.2.9.2) [10] and Mutect2 (v.4.0) [11]. Tumour mutation burden (TMB) was calculated as the total number of all somatic single-nucleotide variants and short insertion/deletions observed in a sample divided by the size of the capture region (67Mb). A threshold for including variants was chosen based on a minimum depth (50 reads) and a minimum variant allele frequency of 10 % as previously published [5]. Mutational signature profiles were calculated using the simulated annealing method previously described by SignatureEstimation [12] using a reduced set of 16 SBS signatures (**Supplementary Table 1**) as previously determined to be present in the colon/colorectal cancer tissue [[5], [6], [7],[13], [14], [15], [16], [17], [18], [19], [20]]. The following RefSeq transcripts were used: NM_001128425.1 (*MUTYH*), NM_002528.7 (*NTHL1*)*,* NM_001369786.1 (*KRAS*) and NM_006218.4 (*PIK3CA*).

The mutational signature profiles of 12 CRCs from eight biallelic *MUTYH* cases and four CRCs from four monoallelic *MUTYH* cases described above have been reported previously [5,6]. Two CRCs from two biallelic cases and two CRCs from two monoallelic *NTHL1* cases described above have been reported previously [7].

### Statistical analysis

For each signature profile, we compared the biallelic cases with the corresponding CRCs or adenomas from the non-hereditary group. Statistical significance between two groups was determined using a two-sided *t-test* with a *p-value*<0.05 considered to be statistically significant. Adjusted *p-values* were calculated using Bonferroni correction for multiple hypothesis testing. For group comparisons, one-way *ANOVA* was used. Additionally, we determined the *Cohen's* d effect size to measure the difference between the means of two subgroups.

### Source code

All data analysis was performed using Python v.3.11 [21], Numpy v.1.24 [22] and Scikit-Learn v.1.3 [23]. Data visualisation was done using the R programming language v.4.3.2 [24] and RStudio v.0.16.0 [25] using the following packages: ggplot2 v.3.5.1 [26], cowplot v.1.1.3 [27] and dplyr v.1.1.4 [28].

## Results

**Fig. 1:**
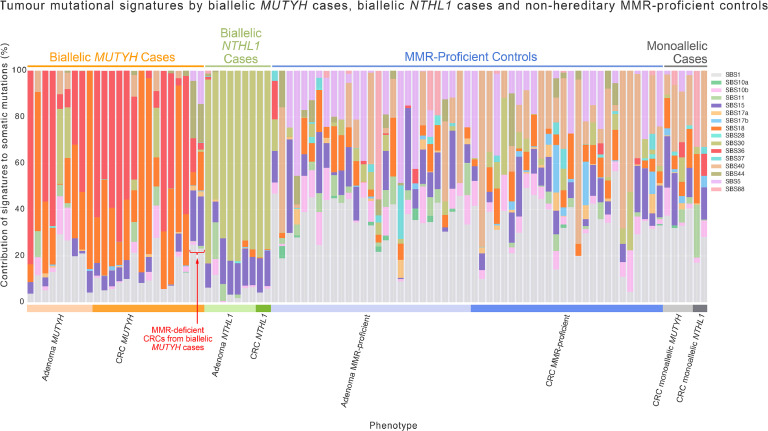
Mutational signatures observed across the colorectal cancers and adenomas included in this study. *Abbreviations: MMR, DNA mismatch repair; SBS, single base substitution; CRC,colorectal cancer.*

The clinicopathological characteristics of the participants and their adenomas and CRCs are shown in Table 1. The biallelic *MUTYH* and biallelic *NTHL1* cases are presented in **Supplementary Table 2**. Of note, all adenomas and CRCs were MMR-proficient by immunohistochemistry except for two biallelic *MUTYH* cases; Pat_301 (2xCRCs at 50 years, one MMR-proficient and one MMR-deficient with MLH1/PMS2 loss), and Pat_315 (1xCRC at 39 years with MSH2/MSH6 loss). The SBS mutational signature profiles of each adenoma and CRC included in the study are presented in Fig. 1.

**Table 1:** The clinicopathological characteristics of the participants and their adenomas and CRCs from each of the biallelic MUTYH cases, biallelic NTHL1 cases and the adenomas and CRCs from the non-hereditary (control) groups included in this study. Overview of the phenotypes by sex, age at diagnosis (including mean and standard deviation), anatomical site, histological type, T stage, grade of tumour and study separated by adenoma and colorectal cancer tissue type and case subgroups.

	*MUTYH* cases (*n* = 13)		*NTHL1* cases (*n* = 5)		Non-hereditary Controls (*n* = 46)		Total (*n* = 64) Individuals
	Adenoma biallelic *MUTYH* (*n* = 9, 10.5 %)	CRC biallelic *MUTYH* (*n* = 15, 17.4 %)	Adenoma biallelic *NTHL1* (*n* = 7, 8.1 %)	CRC biallelic *NTHL1* (*n* = 2, 2.3 %)	MMR-proficient Adenomas (*n* = 27, 31.4 %)	MMR-proficient CRCs (*n* = 26, 30.2 %)	Total (*n* = 86, 100 %) Tissues
Sex, n (%)							
Male	5 (55.6 %)	11 (73.3 %)	3 (42.9 %)	0 (0.0 %)	12 (44.4 %)	12 (46.2 %)	43 (50.0 %)
Female	4 (44.4 %)	4 (26.7 %)	4 (57.1 %)	2 (100.0 %)	15 (55.6 %)	14 (53.8 %)	43 (50.0 %)
Age at diagnosis, n (%)							
Mean ± SD	52.3 ± 14.4	52.2 ± 11.1	57.7 ± 5.7	68.5 ± 10.6	43.7 ± 10.6	42.8 ± 9.1	48.0 ± 12.1
Min. - Max.	33 - 73	33 - 64	51 - 66	61 - 76	27 - 61	21 - 59	21 - 76
≤50 years	2 (22.2 %)	6 (40.0 %)	0 (0.0 %)	0 (0.0 %)	19 (70.4 %)	22 (84.6 %)	49 (57.0 %)
>50 years	7 (77.8 %)	9 (60.0 %)	7 (100.0 %)	2 (100.0 %)	8 (29.6 %)	4 (15.4 %)	37 (43.0 %)
Ancestry, n (%)							
European	9 (100.0 %)	15 (100.0 %)	7 (100.0 %)	2 (100.0 %)	27 (100.0 %)	24 (92.3 %)	84 (97.7 %)
East Asian	0 (0.0 %)	0 (0.0 %)	0 (0.0 %)	0 (0.0 %)	0 (0.0 %)	1 (3.8 %)	1 (1.2 %)
South Asian	0 (0.0 %)	0 (0.0 %)	0 (0.0 %)	0 (0.0 %)	0 (0.0 %)	1 (3.8 %)	1 (1.2 %)
Anatomical site, n (%)							
Caecum	1 (11.1 %)	4 (26.7 %)	0 (0.0 %)	0 (0.0 %)	3 (11.1 %)	3 (11.5 %)	11 (12.8 %)
Ascending	2 (22.2 %)	8 (53.3 %)	1 (14.3 %)	1 (50.0 %)	3 (11.1 %)	3 (11.5 %)	18 (20.9 %)
Transverse	0 (0.0 %)	0 (0.0 %)	1 (14.3 %)	1 (50.0 %)	3 (11.1 %)	6 (23.1 %)	11 (12.8 %)
Descending	0 (0.0 %)	0 (0.0 %)	0 (0.0 %)	0 (0.0 %)	1 (3.7 %)	1 (3.8 %)	2 (2.3 %)
Sigmoid	0 (0.0 %)	1 (6.7 %)	0 (0.0 %)	0 (0.0 %)	5 (18.5 %)	7 (26.9 %)	13 (15.1 %)
Rectum	0 (0.0 %)	2 (13.3 %)	1 (14.3 %)	0 (0.0 %)	6 (22.2 %)	6 (23.1 %)	15 (17.4 %)
Unknown	6 (66.7 %)	0 (0.0 %)	4 (57.1 %)	0 (0.0 %)	6 (22.2 %)	0 (0.0 %)	16 (18.6 %)
Colorectal Adenoma Histological Type, n (%)							
Tubular adenoma	2 (22.2 %)	–	5 (71.4 %)	–	14 (51.9 %)	–	–
Tubulovillous adenoma	4 (44.4 %)	–	2 (28.6 %)	–	13 (48.1 %)	–	–
Unknown	3 (33.3 %)	–	0 (0.0 %)	–	0 (0.0 %)	–	–
CRC Histological Type, n (%)							
Adenocarcinoma	–	15 (100.0 %)	–	2 (100.0 %)	–	24 (92.3 %)	–
Mucinous adenocarcinoma	–	0 (0.0 %)	–	0 (0.0 %)	–	0 (0.0 %)	–
Signet ring adenocarcinoma	–	0 (0.0 %)	–	0 (0.0 %)	–	1 (3.8 %)	–
Undifferentiated (incl. medullary)	–	0 (0.0 %)	–	0 (0.0 %)	–	1 (3.8 %)	–
Grade of CRC, n (%)							
Well differentiated	–	1 (6.7 %)	–	0 (0.0 %)	–	3 (11.5 %)	–
Moderately differentiated	–	13 (86.7 %)	–	2 (100.0 %)	–	16 (61.5 %)	–
Poorly differentiated	–	0 (0.0 %)	–	0 (0.0 %)	–	4 (15.4 %)	–
Unknown	–	1 (6.7 %)	–	0 (0.0 %)	–	3 (11.5 %)	–
Study, n (%)							
ANGELS	0 (0.0 %)	0 (0.0 %)	4 (57.1 %)	0 (0.0 %)	0 (0.0 %)	0 (0.0 %)	4 (4.7 %)
CCFR	6 (66.7 %)	14 (93.3 %)	0 (0.0 %)	1 (50.0 %)	27 (100.0 %)	27 (103.8 %)	74 (86.0 %)
GCPS	3 (33.3 %)	1 (6.7 %)	3 (42.9 %)	1 (50.0 %)	0 (0.0 %)	0 (0.0 %)	8 (9.3 %)

Abbreviations: CRC, colorectal cancer; SD, standard deviation; ANGELS, Applying Novel Genomic approaches to Early-onset and suspected Lynch Syndrome colorectal and endometrial cancers; CCFR, Colon Cancer Family Registry; GCPS, The Genetics of Colonic Polyposis Study.

### The SBS18+SBS36 mutational signature is elevated in both adenomas and CRCs from biallelic MUTYH cases

**Fig. 2:**
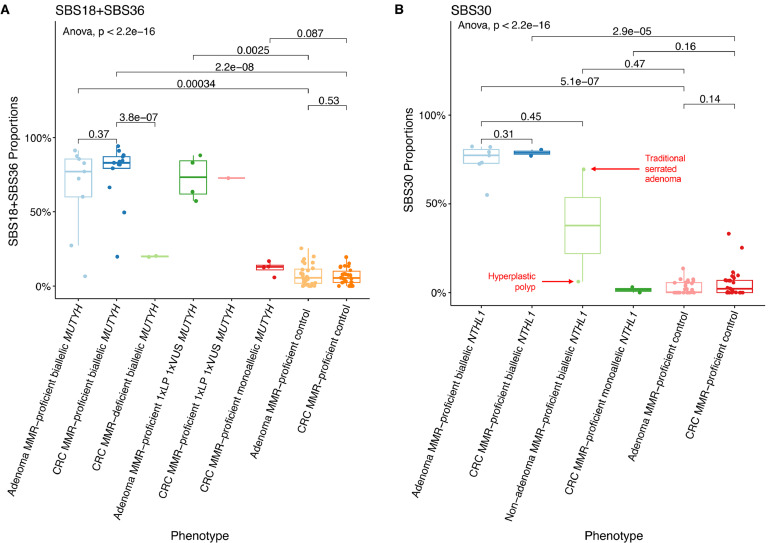
Boxplots of whole-exome sequencing derived genomic features for A) SBS18+SBS36 proportions in *MUTYH* cases and non-hereditary groups and B) SBS30 proportions in *NTHL1* cases and non-hereditary groups. *Abbreviations: MMR, DNA mismatch repair; SBS, single base substitution; CRC,colorectal cancer; VUS, variant of uncertain significance; LP, likely pathogenic.*

**Fig. 3:**
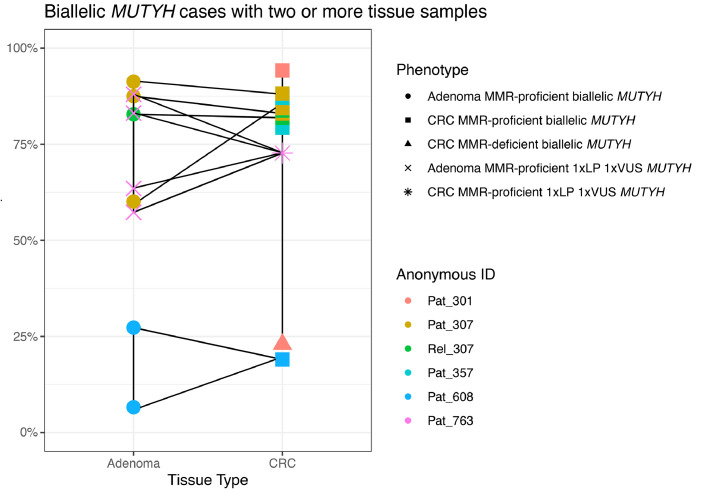
Line plot displaying the comparison of SBS18+SBS36 signature proportions for adenomas and colorectal cancers related to each biallelic *MUTYH* case and for the participant with a pathogenic and variant of uncertain significance in *MUTYH* (Pat_763). *Abbreviations: SBS, single base substitution; CRC, colorectal cancer; VUS, variant of uncertain significance; LP, likely pathogenic; ID, identification; Pat, patient ID; Rel, relative ID.*

The mean (±standard deviation) proportion of SBS18+SBS36 in the adenomas (65.6 %±29.6 %) and MMR-proficient CRCs (76.2 %±20.5 %) from biallelic *MUTYH* cases were not significantly different (*p-value*=0.37) (Fig. 2**A,** Table 2). This result is further highlighted when comparing the SBS18+SB36 proportions in adenomas and CRCs from the same participant (Fig. 3). In contrast, the mean proportion of SBS18+SBS36 in adenomas and CRCs from biallelic *MUTYH* cases were significantly higher compared with the mean proportion in non-hereditary adenomas (65.6 %±29.6 % versus 7.6 %±7.0 %, *p-value*=3.4 × 10^–4^) and CRCs (76.2 %±20.5 % versus 6.5 %±5.5 %, *p-value*=2.2 × 10^–8^) (Fig. 2**A and** Table 3).

**Table 2:** The mean, standard deviation, and range of five genomic features derived from whole-exome sequencing testing and their differences between tissue type and by MUTYH or NTHL1 case or non-hereditary status. Statistically significant *p-values* are highlighted in bold.

	Biallelic *MUTYH* cases					Biallelic *NTHL1* cases					Proficient Controls					
	Adenoma biallelic *MUTYH* (*n* = 9, 10.7 %)	CRC biallelic *MUTYH* (*n* = 13, 15.5 %) 1	*p-value*^2^	Adjusted *p-value*^3^	Effect Size (Cohen's d)	Adenoma biallelic *NTHL1* (*n* = 7, 8.3 %)	CRC biallelic *NTHL1* (*n* = 2, 2.4 %)	*p-value*^2^	Adjusted *p-value*^3^	Effect Size (Cohen's d)	Adenoma proficient control (*n* = 27, 32.1 %)	CRC proficient control (*n* = 26, 31.0 %)	*p-value*^2^	Adjusted *p-value*^3^	Effect Size (Cohen's d)	Total (*n* = 84, 100 %)
SBS18+SBS36			0.37	1.00	−0.4			0.30	1.00	0.5			0.53	1.00	0.2	
Mean	65.6 %	76.2 %				1.0 %	0.4 %				7.6 %	6.5 %				23.4 %
SD	29.6 %	20.5 %				1.4 %	0.0 %				7.0 %	5.5 %				32.0 %
Range	6.6 % - 91.3 %	19.8 % - 94.2 %				0 % - 3.2 %	0.4 % - 0.4 %				0 % - 25.4 %	0 % - 19.5 %				0 % - 94.2 %
SBS30			0.38	1.00	0.4			0.31	1.00	−0.5			0.14	1.00	−0.4	
Mean	6.0 %	2.0 %				74.5 %	78.8 %				2.8 %	5.4 %				11.6 %
SD	12.3 %	6.0 %				9.4 %	2.4 %				3.6 %	8.0 %				23.4 %
Range	0 % - 32.4 %	0 % - 21.4 %				55 % - 82.3 %	77.1 % - 80.5 %				0 % - 13.6 %	0 % - 33.2 %				0 % - 82.3 %
TMB			0.12	1.00	−0.8			0.87	1.00	−0.1			**7.4 × 10^–5^**	**2.6 × 10^–3^**	−1.2	
Mean	4.8	7.5				7.1	7.5				1.5	2.8				3.8
SD	4.3	2.9				4.2	2.0				0.9	1.3				3.3
Range	0.3 - 12.7	3.5 - 13.3				2.5 - 14.8	6.1 - 8.8				0.3 - 4.1	1.4 - 5.9				0.3 - 14.8
INDEL count			**0.02**	0.77	−1.1			0.95	1.00	0.0			**2.3 × 10^–3^**	0.08	−0.9	
Mean	4.4	9.7				10.7	10.5				7.1	11.3				8.9
SD	4.9	4.6				9.3	0.7				4.2	5.4				5.6
Range	1 - 17	2 −16				2 - 25	10 - 11				1 - 17	3 - 20				1 - 25
SNV count			0.13	1.00	−0.7			0.87	1.00	−0.1			**9.8 × 10^–5^**	**3.4 × 10^–3^**	−1.2	
Mean	318.6	494.3				466.9	491.0				91.1	178.1				245.6
SD	285.4	197.5				274.5	132.9				56.8	86.9				217.9
Range	19 - 851	231 - 895				168 - 974	397 - 585				16 - 262	84 - 381				16 - 974

Abbreviations: CRC, colorectal cancer; SBS, single base substitution; SD, standard deviation; TMB, tumour mutation burden; INDEL, large insertion/deletion; SNV, single nucleotide variant.

1 MMR-proficient CRCs only from biallelic MUTYH cases were included.

2 Two-tailed *t*-test.

3 *p-values* were calculated using the Bonferroni correction to adjust for multiple (*n* = 35) hypothesis testing.

**Table 3:** The mean, standard deviation, and range of five genomic features derived from whole-exome sequencing testing assessed for their differences between MUTYH or NTHL1 case or non-hereditary status for colorectal adenomas and colorectal cancers separately. Statistically significant *p-values* are highlighted in bold.

	Colorectal Adenomas					Colorectal Cancers					Colorectal Adenomas					Colorectal Cancers					
	Biallelic *MUTYH* cases (*n* = 9, 10.7 %)	Non-hereditary controls (*n* = 27, 32.1 %)	*p-value*^2^	Adjusted *p-value*^3^	Effect Size (Cohen's d)	Biallelic *MUTYH* cases (*n* = 13, 15.5 %) 1	Non-hereditary controls (*n* = 26, 31.0 %)	*p-value*^2^	Adjusted *p-value*^3^	Effect Size (Cohen's d)	Biallelic *NTHL1* cases (*n* = 7, 8.3 %)	Non-hereditary controls (*n* = 27, 32.1 %)	*p-value*^2^	Adjusted *p-value*^3^	Effect Size (Cohen's d)	Biallelic *NTHL1* cases (*n* = 2, 2.4 %)	Non-hereditary controls (*n* = 26, 31.0 %)	*p-value*^2^	Adjusted *p-value*^3^	Effect Size (Cohen's d)	Total (*n* = 84, 100 %)
SBS18+SBS36			**3.4 × 10^–4^**	**1.2 × 10^–2^**	3.7			**2.2 × 10^–8^**	**7.7 × 10^–7^**	5.6			**8.0 × 10^–5^**	**2.8 × 10^–3^**	−1.0			**6.4 × 10^–6^**	**2.2 × 10^–4^**	−1.1	
Mean	65.6 %	7.6 %				76.2 %	6.5 %				1.0 %	7.6 %				0.4 %	6.5 %				23.4 %
SD	29.6 %	7.0 %				20.5 %	5.5 %				1.4 %	7.0 %				0.0 %	5.5 %				32.0 %
Range	6.6 % - 91.3 %	0 % - 25.4 %				19.8 % - 94.2 %	0 % - 19.5 %				0 % - 3.2 %	0 % - 25.4 %				0.4 % - 0.4 %	0 % - 19.5 %				0 % - 94.2 %
SBS30			0.45	1.00	0.5			0.15	1.00	−0.5			**5.1 × 10^–7^**	**1.8 × 10^–5^**	13.8			**2.9 × 10^–5^**	**1.0 × 10^–3^**	9.3	
Mean	6.0 %	2.8 %				2.0 %	5.4 %				74.5 %	2.8 %				78.8 %	5.4 %				11.6 %
SD	12.3 %	3.6 %				6.0 %	8.0 %				9.4 %	3.6 %				2.4 %	8.0 %				23.4 %
Range	0 % - 32.4 %	0 % - 13.6 %				0 % - 21.4 %	0 % - 33.2 %				55 % - 82.3 %	0 % - 13.6 %				77.1 % - 80.5 %	0 % - 33.2 %				0 % - 82.3 %
TMB			**4.7 × 10^–2^**	1.00	1.5			**7.5 × 10^–5^**	**2.6 × 10^–3^**	2.3			**1.2 × 10^–2^**	0.42	2.8			0.18	1.00	3.4	
Mean	4.8	1.5				7.5	2.8				7.1	1.5				7.5	2.8				3.8
SD	4.3	0.9				2.9	1.3				4.2	0.9				2.0	1.3				3.3
Range	0.3 - 12.7	0.3 - 4.1				3.5 - 13.3	1.4 - 5.9				2.5 - 14.8	0.3 - 4.1				6.1 - 8.8	1.4 - 5.9				0.3 - 14.8
INDEL count			0.18	1.00	−0.6			0.33	1.00	−0.3			0.35	1.00	0.7			0.48	1.00	−0.2	
Mean	4.4	7.1				9.7	11.3				10.7	7.1				10.5	11.3				8.9
SD	4.9	4.2				4.6	5.4				9.3	4.2				0.7	5.4				5.6
Range	1 - 17	1 - 17				2 - 16	3 - 20				2 - 25	1 - 17				10 - 11	3 - 20				1 - 25
SNV count			**4.4 × 10^–2^**	1.00	1.5			**6.9 × 10^–5^**	**2.4 × 10^–3^**	2.4			**1.1 × 10^–2^**	0.39	2.9			0.18	1.00	3.5	
Mean	318.6	91.1				494.3	178.1				466.9	91.1				491.0	178.1				245.6
SD	285.4	56.8				197.5	86.9				274.5	56.8				132.9	86.9				217.9
Range	19 - 851	16 - 262				231 - 895	84 - 381				168 - 974	16 - 262				397 - 585	84 - 381				16 - 974

Abbreviations: CRC, colorectal cancer; SBS, single base substitution; SD, standard deviation; TMB, tumour mutation burden; INDEL, large insertion/deletion; SNV, single nucleotide variant.

1 MMR-proficient CRCs only from biallelic MUTYH cases were included.

2 Two-tailed *t*-test.

3 p-values were calculated using the Bonferroni correction to adjust for multiple (*n* = 35) hypothesis testing.

### Co-occurrence of mutational processes related to defective MUTYH and defective DNA mismatch repair

**Fig. 4:**
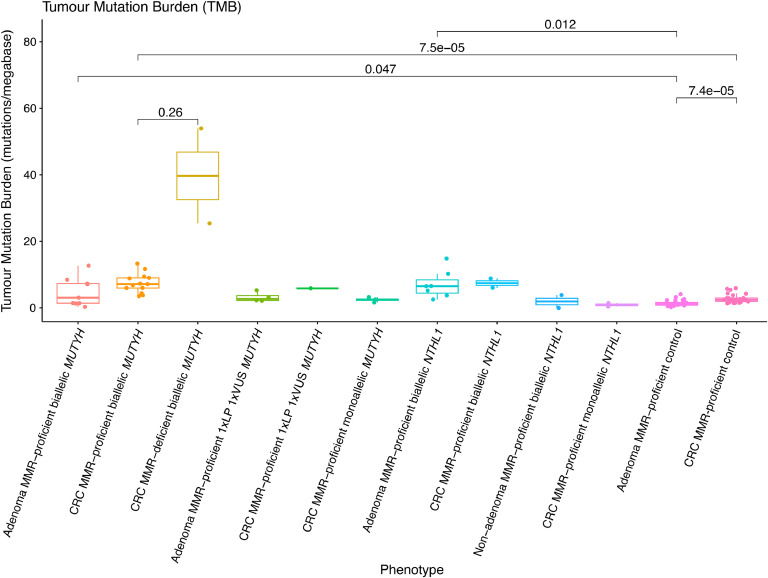
Boxplots of whole-exome sequencing derived genomic features for tumour mutation burden across the adenomas and CRCs from the MUTYH, NTHL1 and non-hereditary groups. Red line indicates threshold for hypermutation status (≥10 mutations / mega base). Except for the comparison of MMR-deficient biallelic MUTYH cases with MMR-proficient biallelic MUTYH cases, all other comparisons were not significant (p-value≥0.05). Abbreviations: TMB, tumour mutation burden; MMR, DNA mismatch repair; CRC, colorectal cancer; VUS, variant of uncertain significance; LP, likely pathogenic.

In the two MMR-deficient CRCs from biallelic *MUTYH* cases (Pat_301 and Pat_315), the mean proportion of SBS18+SBS36, was significantly lower compared with the MMR-proficient CRCs from biallelic *MUTYH* cases (20.0 % ± 0.5 % versus 76.2 % ± 20.5 %, *p-value*=3.8 × 10^–7^) but they were still higher compared with non-hereditary CRCs (6.5 % ± 5.5 %, *p-value*=2.2 × 10^–8^) (Fig. 2**A and** Table 3). Both these MMR-deficient CRCs also showed higher proportions of SBS15 and SBS44, which are mutational signatures associated with MMR-deficiency (Fig. 1). In addition, the TMB of these two MMR-deficient CRCs (53.9 and 25.4 mutations/Mb, respectively) was higher compared with the mean TMB of the MMR-proficient CRCs from biallelic *MUTYH* cases (7.5 ± 2.9 mutations/Mb) (Fig. 4).

The cause of MMR-deficiency in Pat_301 and Pat_315 was not related to carrying a germline LP/P variant in one of the DNA MMR genes, but rather from two somatic MMR mutations causing biallelic inactivation in each CRC as determined from the WES data. The CRC showing loss of MLH1/PMS2 protein expression from Pat_301 had two somatic mutations in *MLH1* (c.1813G>*T* p.(Glu605Ter) and c.1816G>*T* p.(Gly606Ter)) and no evidence of tumour *MLH1* promoter hypermethylation. The CRC showing loss of MSH2/MSH6 protein expression from Pat_315 had a somatic mutation in *MSH2* (c.394G>*T* p.(Glu132Ter)) and loss of heterozygosity indicating loss of the wildtype *MSH2* allele. The somatic single nucleotide mutations observed in *MLH1* and *MSH2* matched the mutational contexts associated with SBS18 and SBS36 (Pat_301:TCT>TAT and TCC>TAC; Pat_315:TCA>TTA), suggesting the constitutionally defective *MUTYH* contributed to these somatic MMR mutational events and resulted in MMR-deficiency in these two CRCs. Interestingly, the synchronous MMR-proficient CRC from Pat_301 exhibited a high proportion of SBS18+SBS36 (94.2 %) and a low TMB (9.4 mutations/Mb), further highlighting the impact of tumour MMR-deficiency on the SBS18+SBS36 signature proportions in biallelic *MUTYH* cases.

### Somatic mutations as biomarkers of biallelic MUTYH status in adenomas

Previously, the *KRAS* c.34G>*T* p.(Gly12Cys) and *PIK3CA* c.1636C>A p.(Gln546Lys) somatic mutations were shown to be recurrent mutations significantly increased in CRCs from biallelic *MUTYH* pathogenic variant cases [6]. In adenomas, the *KRAS* c.34G>*T* mutation was present in 6/9 (66.7 %) and 2/27 (7.4 %) of the biallelic *MUTYH* and non-hereditary adenomas, respectively (*p-value*=3.1 × 10^–2^). The *KRAS* c.34G>*T* mutation had a positive predictive value of 75 % and a negative predictive value of 89.3 % in adenomas compared with a positive predictive value of 100 % and negative predictive value of 86.7 % in CRCs, indicating that the somatic *KRAS* mutation may not be as clinically useful in identifying biallelic *MUTYH* cases in adenomas as it is in CRCs. The *PIK3CA* c.1636C>A mutation was not observed in adenomas from biallelic *MUTYH* cases or in adenomas from the non-hereditary group (**Supplementary Figure 1**).

### The SBS18+SBS36 mutational signature provides evidence for variant classification

We profiled four adenomas and a CRC from Pat_763 who carried a germline heterozygous pathogenic variant (c.1187G>*A* p.(Gly396Asp)) and a germline heterozygous VUS (c.533G>C p.(Gly178Ala)) in *MUTYH.* All four adenomas (mean proportion: 73.0 %±14.9 %, range: 57.3 %−88.0 %) and the CRC (72.7 %) demonstrated high proportions of SBS18+SBS36 consistent with germline biallelic inactivation of *MUTYH* (Figs. 2**A and** Fig. 3). No somatic second hits in *MUTYH* were observed that may have accounted for the high SBS18+SBS36 signature proportions in the adenomas and CRC. These findings support a reclassification of the *MUTYH* c.533G>C p.(Gly178Ala) variant as likely pathogenic.

### The SBS30 mutational signature is elevated in both adenomas and CRCs from biallelic NTHL1 cases

The mean proportion of SBS30 in adenomas (74.5 %±9.4 %) and CRCs (78.8 %±2.4 %) from biallelic *NTHL1* cases were not significantly different (*p-value*=0.31) (Fig. 2**B and** Table 2). The mean proportion of SBS30 in adenomas from biallelic *NTHL1* cases was, however, significantly higher compared with the mean proportion in non-hereditary adenomas (74.5 %±9.4 % versus 2.8 %±1.3 %; p-value=5.1 × 10^–7^) (Fig. 2**B and** Table 3). In addition to 7 adenomas and 2 CRCs, a hyperplastic polyp and a traditional serrated adenoma from two biallelic *NTHL1* cases (Pat_005 and Pat_469) were tested. Of note, the traditional serrated adenoma showed high proportion of SBS30 at 69.4 %, whereas the SBS30 proportion in the hyperplastic polyp was only 6.2 % (Fig. 2**B**).

## Discussion

In this study, we showed that the SBS18+SBS36 and SBS30 mutational signatures associated with biallelic *MUTYH* and biallelic *NTHL1* deficiencies, were present in adenomas at similar proportions to those observed in CRCs and were significantly higher when compared with the proportions observed in non-hereditary adenomas and CRCs. Together, these results demonstrate that the presence of these mutational processes and consequent mutational signatures, at diagnostic levels in the pre-malignant stage are equivalent to levels observed in tumours. Thus, testing of precancerous adenomas could expand the potential tissue available for profiling. This is particularly important in cases where tumour DNA may not be available for testing or where the individual has not yet developed a cancer. Being able to identify high-risk individuals at an early stage who may benefit from tailored screening, treatment and prevention strategies, is imperative for improved CRC detection and prevention. Identification of biallelic *MUTYH* or biallelic *NTHL1* cases may facilitate prevention of non-CRC cancers through screening or prophylactic surgery.

A previous study by Grolleman et al. has shown that the mutation spectrum in biallelic *NTHL1* carriers can predispose to a wider tumour spectrum than just affecting the colon and includes tumours of the breast, endometrial, head and neck squamous cell carcinoma, meningioma, thyroid and urothelial cell cancers [7]. They further showed that SBS30 was the main mutational process in these tumours [7], highlighting that different tissue sources can be used to determine biallelic *NTHL1*-deficiency.

We identified two scenarios where SBS30 or SBS18+SBS36 may present with limitations. Firstly, although SBS30 was shown to be a predominant mutational signature in adenomas from biallelic *NTHL1* cases, our results showed variable presence of SBS30 in two serrated polyp subtypes, 69.4 % in the traditional serrated adenoma and only 6.2 % in the hyperplastic polyp. As biallelic *NTHL1* cases can present with mixed polyp types [29], further research is needed to determine the utility of testing serrated polyps for mutational signatures for *NTHL1* and more broadly for other hereditary CRC/polyposis syndromes. While the proposal of using adenomas as a surrogate biomarker for CRC is not new, suggestions of prioritising advanced adenomas (≥1 cm) over small adenomas have been made [30]. Large adenomas are more reflective of a tumour's molecular profile and are more likely to progress to cancer than small adenomas [30]. For example, adenomas with dysplasia, a feature of advanced adenomas, are more likely to demonstrate loss of MMR protein expression in people with Lynch syndrome than adenomas without dysplasia [31]. Hence, if given a choice, large and/or advanced adenomas may be preferred for testing.

Secondly, we tested two MMR-deficient CRCs from two biallelic *MUTYH* cases where the mutational signature profile showed defective MMR related to the presence of SBS15 and SBS44 and a hypermutated TMB that co-occurred with the SBS18+SBS36 signature, albeit at lower proportions than observed in MMR-proficient CRCs from biallelic *MUTYH* cases. These findings highlight MMR-deficiency as an important diagnostic caveat for utilising SBS18+SBS36 to identify biallelic *MUTYH* cases or for classifying variants.

This study extends on our previous work for applying SBS18+SBS36 in CRCs to reclassify VUSs in *MUTYH* [5]. We showed high levels of SBS18+SBS36 in the CRC and multiple adenomas from the same person provides high confidence that the *MUTYH* c.533G>C p.(Gly178Ala) variant is pathogenic. Additional evidence, including its absence in gnomAD and *in-silico* predictions from REVEL, SIFT, PolyPhen-2 and Align-GVGD suggest this missense change affects protein function, further supporting pathogenicity (https://www.ncbi.nlm.nih.gov/clinvar/variation/481808/, last accessed date: August 1st, 2024). The ability to test multiple independent adenomas/CRCs provides high confidence for variant classification where all or none of the lesions have the signature. The clinical genetics community is increasingly challenged by VUS, where almost half (47.8 %, 1329/2782) of the *MUTYH* variants in ClinVar are currently classified as VUS (last accessed on: August 6th, 2024) [32]. Approaches to classify variants with existing and widely used infrastructure i.e., next generation sequencing and validated bioinformatic tools, will aid in reclassifying variants and optimising clinical management and cancer prevention for the patient and their relatives.

Limitations of this study include the lack of ancestry diversity within the case and non-hereditary groups which were predominantly white European. Similarly, there was a limited range of germline LP/P variants for both *MUTYH* and *NTHL1*. The consistency of mutational signature findings across a broader group of cases of different pathogenic variants and ancestry would provide evidence of the robustness of this approach. All of the CRCs and adenomas tested in this study were from FFPE tissue, however we have previously shown that mutational signature profiling is effective in both FFPE and fresh frozen tissue DNA samples [5].

## Conclusions

This study provides important findings demonstrating that testing adenomas for SBS18+SBS36 or SBS30 can be an equally effective alternative to identifying biallelic *MUTYH* or biallelic *NTHL1* cases, respectively, if CRC has not yet developed or tissue is not available. This provides important opportunities for clinical management decision-making such as colectomy versus endoscopic polypectomy for CRC prevention given the established high CRC penetrance in biallelic cases. Furthermore, the specificity of these signatures enables the utility of mutational signature profiling to classify VUS. Our study identified potential caveats to using mutational signatures diagnostically, namely, the presence of MMR-deficiency which may diminish the SBS18+SBS36 signature, while for SBS30, testing of serrated polyps needs further investigation. This study adds to the growing evidence of the clinical utility of gene specific mutational signature profiling for identifying hereditary CRC/polyposis syndromes and further expands the opportunities to utilise mutational signatures as a supportive feature for variant classification.

## Availablility of data and materials

The datasets used and/or analysed during the current study are available from the corresponding author on reasonable request.

## Funding

Funding by a 10.13039/501100000925National Health and Medical Research Council of Australia (NHMRC) Investigator grant GNT1194896 awarded to DDB and funding by a Cure Cancer Early Career Research Grant awarded to PG supported the design, analysis, and interpretation of data. DDB is supported by a University of Melbourne Dame Kate Campbell Fellowship. PG is supported by an NHMRC Investigator Grant (GNT2026331). RW is supported by the University of Melbourne Early Career Researcher Grant. BJP is supported by a Victorian Health and Medical Research Fellowship from the Victorian Government. 10.13039/501100007066AKW is supported by an NHMRC Investigator grant (GNT1194392). JLH is supported by the University of Melbourne Dame Kate Campbell Fellowship. MAJ is supported by an NHMRC Investigator grant (GNT1195099).

The Colon Cancer Family Registry (CCFR, www.coloncfr.org) is supported in part by funding from the National Cancer Institute (NCI), National Institutes of Health (NIH) (award U01 CA167551). Support for case ascertainment was provided in part from the Surveillance, Epidemiology, and End Results (SEER) Program and the following U.S. state cancer registries: AZ, CO, MN, NC, NH; and by the Victoria Cancer Registry (Australia) and Ontario Cancer Registry (Canada). The content of this manuscript does not necessarily reflect the views or policies of the NIH or any of the collaborating centres in the CCFR, nor does mention of trade names, commercial products, or organisations imply endorsement by the US Government, any cancer registry, or the CCFR.

## CRediT authorship contribution statement

**Romy Walker:** Writing – review & editing, Writing – original draft, Visualization, Validation, Software, Methodology, Investigation, Funding acquisition, Formal analysis, Data curation, Conceptualization. **Jihoon E. Joo:** Writing – review & editing, Visualization, Software, Investigation, Formal analysis. **Khalid Mahmood:** Writing – review & editing, Software, Investigation. **Mark Clendenning:** Writing – review & editing, Investigation. **Julia Como:** Writing – review & editing, Investigation. **Susan G. Preston:** Writing – review & editing, Investigation. **Sharelle Joseland:** Writing – review & editing, Resources. **Bernard J. Pope:** Writing – review & editing, Software, Funding acquisition. **Ana B.D. Medeiros:** Writing – review & editing, Resources, Formal analysis. **Brenely V. Murillo:** Writing – review & editing, Resources. **Nicholas Pachter:** Writing – review & editing, Resources. **Kevin Sweet:** Writing – review & editing, Resources. **Allan D. Spigelman:** Writing – review & editing, Resources. **Alexandra Groves:** Writing – review & editing, Resources. **Margaret Gleeson:** Writing – review & editing, Resources. **Krzysztof Bernatowicz:** Writing – review & editing, Resources. **Nicola Poplawski:** Writing – review & editing, Resources. **Lesley Andrews:** Writing – review & editing, Resources. **Emma Healey:** Writing – review & editing, Resources. **Steven Gallinger:** Writing – review & editing, Resources. **Robert C. Grant:** Writing – review & editing, Resources. **Aung K. Win:** Writing – review & editing, Resources. **John L. Hopper:** Writing – review & editing, Resources. **Mark A. Jenkins:** Writing – review & editing, Resources. **Giovana T. Torrezan:** Writing – review & editing, Resources, Data curation, Conceptualization. **Christophe Rosty:** Writing – review & editing, Data curation. **Finlay A. Macrae:** Writing – review & editing, Resources. **Ingrid M. Winship:** Writing – review & editing, Resources. **Daniel D. Buchanan:** Writing – review & editing, Validation, Supervision, Resources, Project administration, Methodology, Investigation, Funding acquisition, Data curation, Conceptualization. **Peter Georgeson:** Writing – review & editing, Visualization, Validation, Supervision, Software, Methodology, Investigation, Funding acquisition, Formal analysis, Data curation, Conceptualization.

## Declaration of competing interest

The authors declare the following financial interests/personal relationships which may be considered as potential competing interests:

Robert C. Grant received a scholarship from Pfizer and provided consulting or advisory roles for Astrazeneca, Tempus, Eisai, Incyte, Knight Therapeutics, Guardant Health, and Ipsen. All other authors have no relevant financial or non-financial interests to disclose.

## Appendix Supplementary materials

Image, application 1

Image, application 2
