# Supplementary material for: Adenomas from individuals with pathogenic biallelic variants in the MUTYH and NTHL1 genes demonstrate base excision repair tumour mutational signature profiles similar to colorectal cancers, expanding potential diagnostic and variant classification applications
Source: Transl Oncol. 2025 Jan 9;52:102266. doi: 10.1016/j.tranon.2024.102266 (PMC11774829; doi:10.1016/j.tranon.2024.102266)
Supplement: Supplementary file 1 [file mmc1.docx]

**Supplementary Files for**

**Adenomas from individuals with pathogenic biallelic variants in the *MUTYH* and *NTHL1* genes demonstrate base excision repair tumour mutational signature profiles similar to colorectal cancers, expanding potential diagnostic and variant classification applications**

Table of Contents

[SUPPLEMENTARY FIGURE 3](#_Toc174106054)

[Supplementary Figure S1. Bar plots displaying the proportion of known hotspot mutations A) *KRAS* c.34G>T p.(Gly12Cys) and B) *PIK3CA* c.1636C>A p.(Gln546Lys) in the adenomas and CRCs in this study 3](#_Toc174106055)

[SUPPLEMENTARY TABLES 4](#_Toc174106056)

[Supplementary Table S1. Reduced set of mutational signatures used in this study and their proposed aetiologies (if known). 4](#_Toc174106057)

[Supplementary Table S2. Participants and their germline *MUTYH* (NM_001128425.1) and *NTHL1* (NM_002528.7) variants identified from clinical diagnostic or research testing included in this study. 5](#_Toc174106058)

# SUPPLEMENTARY FIGURE

**Supplementary Figure S1.** Bar plots displaying the proportion of known hotspot mutations A) *KRAS* c.34G>T p.(Gly12Cys) and B) *PIK3CA* c.1636C>A p.(Gln546Lys) in the adenomas and CRCs in this study. *Abbreviations: MMR, DNA mismatch repair; CRC, colorectal cancer.*

# SUPPLEMENTARY TABLES

## **Supplementary Table S1.** Reduced set of mutational signatures used in this study and their proposed aetiologies (if known).

| # | Signature | Proposed Aetiology | Reason for inclusion | Reference^1^ |
| --- | --- | --- | --- | --- |
| 1 | SBS1 | Spontaneous deamination of 5-methylcytosine (clock-like signature) | Set of signatures observed in 59 whole-exome sequenced CRCs | Alexandrov *et al.*, 2020; Everall *et al.*, 2023 |
| 2 | SBS5 | Unknown (clock-like signature) | Set of signatures observed in 59 whole-exome sequenced CRCs | Alexandrov *et al.*, 2020 |
| 3 | SBS10a | Polymerase epsilon exonuclease domain mutations | Set of signatures observed in 59 whole-exome sequenced CRCs | Alexandrov *et al.*, 2020; Everall *et al.*, 2023 |
| 4 | SBS10b | Polymerase epsilon exonuclease domain mutations | Set of signatures observed in 59 whole-exome sequenced CRCs | Alexandrov *et al.*, 2020; Everall *et al.*, 2023 |
| 5 | SBS11 | Red meat intake; Temozolomide treatment | Observed in individuals with a high unprocessed red meat consumption (high-risk for causing CRC) and observed in CRCs that were treated with Temozolomide (immune-checkpoint inhibitor blockade) | Gurjao *et al.*, 2021; Crisafulli *et al.*, 2022 |
| 6 | SBS15 | Defective DNA mismatch repair | Set of signatures observed in 59 whole-exome sequenced CRCs | Alexandrov *et al.*, 2020; Everall *et al.*, 2023 |
| 7 | SBS17a | Unknown | Set of signatures observed in 59 whole-exome sequenced CRCs | Alexandrov *et al.*, 2020; Everall *et al.*, 2023 |
| 8 | SBS17b | Unknown | Set of signatures observed in 59 whole-exome sequenced CRCs | Alexandrov *et al.*, 2020; Everall *et al.*, 2023 |
| 9 | SBS18 | Damage by reactive oxygen species | Set of signatures observed in 59 whole-exome sequenced CRCs | Alexandrov *et al.*, 2020; Everall *et al.*, 2023 |
| 10 | SBS28 | Unknown | Set of signatures observed in 59 whole-exome sequenced CRCs | Alexandrov *et al.*, 2020; Everall *et al.*, 2023 |
| 11 | SBS30 | Defective base excision repair due to *NTHL1* mutations | Observed in CRC-affected individuals diagnosed with *NTHL1*-associated polyposis | Grolleman *et al.*, 2019 |
| 12 | SBS36 | Defective base excision repair due to *MUTYH* mutation | Observed in CRC-affected individuals diagnosed with *MUTYH*-associated polyposis | Viel *et al.*, 2017, Georgeson *et al.*, 2022 |
| 13 | SBS37 | Unknown | Set of signatures observed in 59 whole-exome sequenced CRCs | Alexandrov *et al.*, 2020 |
| 14 | SBS40 | Unknown | Set of signatures observed in 59 whole-exome sequenced CRCs | Alexandrov *et al.*, 2020 |
| 15 | SBS44 | Defective DNA mismatch repair | Set of signatures observed in 59 whole-exome sequenced CRCs | Alexandrov *et al.*, 2020; Everall *et al.*, 2023 |
| 16 | SBS88 | Colibactin exposure (E. coli bacteria carrying pks pathogenicity island) | Observed in CRCs caused by *Escherichia coli* producing the genotoxin colibactin | Pleguezuelos-Manzano et al., 2020; Everall *et al.*, 2023 |

*Abbreviations: SBS, single base substitution; ID, small insertion/deletion; CRC, colorectal cancer.*

^1^ The mutational signatures and proposed aetiologies listed here are part of the SBS and ID mutational signature spectrum as published by Tate *et al.* (COSMIC v3.2).

## **Supplementary Table S2.** Participants and their germline *MUTYH* (NM_001128425.1) and *NTHL1* (NM_002528.7) variants identified from clinical diagnostic or research testing included in this study.

| # | Patient ID | Study | AgeDx | Sex | MMR | Tissue tested | Gene | Germline variants | State | ClinVar classification | Genotype |
| --- | --- | --- | --- | --- | --- | --- | --- | --- | --- | --- | --- |
| 1 | Pat_307 | CCFR | 62 | M | pMMR | 3x adenoma, 3x CRC | *MUYTH* | c.536A>G p.(Tyr179Cys) | Homozygous | (Likely) pathogenic | Biallelic *MUTYH* case |
| 2 | Rel_307 | CCFR | 56 | F | pMMR | 1x adenoma, 2x CRC | *MUYTH* | c.536A>G p.(Tyr179Cys) | Homozygous | (Likely) pathogenic | Biallelic *MUTYH* case |
| 3 | Pat_231 | CCFR | 54 | M | pMMR | 1x CRC | *MUYTH* | c.545G>A p.(Arg182His) \| c.536A>G p.(Tyr179Cys) | Compound heterozygous | Pathogenic \| (Likely) pathogenic | Biallelic *MUTYH* case |
| 4 | Pat_357 | CCFR | 64 | M | pMMR | 2x CRC | *MUYTH* | c.1187G>A p.(Gly396Asp) | Homozygous | (Likely) pathogenic | Biallelic *MUTYH* case |
| 5 | Pat_608 | CCFR | 33 | M | pMMR | 2x adenoma, 1x CRC | *MUYTH* | c.1147del p.(Ala385ProfsTer23) | Homozygous | (Likely) pathogenic | Biallelic *MUTYH* case |
| 6 | Pat_301 | CCFR | 50 | F | pMMR | 1x CRC | *MUYTH* | c.536A>G p.(Tyr179Cys) | Homozygous | (Likely) pathogenic | Biallelic *MUTYH* case |
| 7 | Pat_301 | CCFR | 50 | F | dMMR/pMMR | 2x CRCs | *MUYTH* | c.536A>G p.(Tyr179Cys) | Homozygous | (Likely) pathogenic | Biallelic *MUTYH* case |
| 8 | Pat_315 | CCFR | 39 | M | dMMR | 1x CRC | *MUYTH* | c.1187G>A p.(Gly396Asp) | Homozygous | (Likely) pathogenic | Biallelic *MUTYH* case |
| 9 | Pat_822 | CCFR | 39 | M | pMMR | 1x CRC | *MUYTH* | c.536A>G p.(Tyr179Cys) \| c.734G>A p.(Arg245His) | Compound heterozygous | (Likely) pathogenic \| (Likely) pathogenic | Biallelic *MUTYH* case |
| 10 | Pat_206 | CCFR | 59 | M | pMMR | 1x CRC | *MUYTH* | c.1187G>A p.(Gly396Asp) | Homozygous | (Likely) pathogenic | Biallelic *MUTYH* case |
| 11 | Pat_041 | GCPS | 33 | M | pMMR | 1x CRC | *MUYTH* | c.1147del p.(Ala385ProfsTer23) | Homozygous | (Likely) pathogenic | Biallelic *MUTYH* case |
| 12 | Pat_509 | GCPS | 65 | F | pMMR | 2x adenoma | *MUYTH* | c.536A>G p.(Tyr179Cys) | Homozygous | (Likely) pathogenic | Biallelic *MUTYH* case |
| 13 | Pat_706 | GCPS | 73 | F | pMMR | 1x adenoma | *MUYTH* | c.1187G>A p.(Gly396Asp) \| c.933+3A>C p.? | Compound heterozygous | (Likely) pathogenic \| Pathogenic | Biallelic *MUTYH* case |
| 14 | Pat_763 | GCPS | 55 | M | pMMR | 4x adenoma, 1x CRC | *MUYTH* | c.1187G>A p.(Gly396Asp) \| c.533G>C p.(Gly178Ala) | Compound heterozygous | Pathogenic \| Variant of uncertain significance | Suspected biallelic *MUTYH* case |
| 15 | Rel_357 | CCFR | 64 | F | pMMR | 1x CRC | *MUYTH* | c.1187G>A p.(Gly396Asp) | Heterozygous | (Likely) pathogenic | Monoallelic *MUTYH* case |
| 16 | Pat_036 | CCFR | 35 | F | pMMR | 1x CRC | *MUYTH* | c.1187G>A p.(Gly396Asp) | Heterozygous | (Likely) pathogenic | Monoallelic *MUTYH* case |
| 17 | Pat_400 | CCFR | 49 | M | pMMR | 1x CRC | *MUYTH* | c.1187G>A p.(Gly396Asp) | Heterozygous | (Likely) pathogenic | Monoallelic *MUTYH* case |
| 18 | Pat_018 | ANGELS | 39 | F | pMMR | 1x CRC | *MUYTH* | c.1187G>A p.(Gly396Asp) | Heterozygous | (Likely) pathogenic | Monoallelic *MUTYH* case |
| 19 | Pat_427 | GCPS | 57 | M | pMMR | 3x adenoma | *NTHL1* | c.244C>T p.(Gln82Ter) | Homozygous | (Likely) pathogenic | Biallelic *NTHL1* case |
| 20 | Pat_445 | ANGELS | 53 | F | pMMR | 2x adenoma | *NTHL1* | c.835C>T p.(Gln279Ter) \| c.244C>T p.(Gln82Ter) | Compound heterozygous | (Likely) pathogenic \| (Likely) pathogenic | Biallelic *NTHL1* case |
| 21 | Rel_445 | ANGELS | 56 | F | pMMR | 2x adenoma | *NTHL1* | c.835C>T p.(Gln279Ter) \| c.244C>T p.(Gln82Ter) | Compound heterozygous | (Likely) pathogenic \| (Likely) pathogenic | Biallelic *NTHL1* case |
| 22 | Pat_469 | GCPS | 76 | F | pMMR | 1x adenoma, 1x CRC | *NTHL1* | c.244C>T p.(Gln82Ter) | Homozygous | (Likely) pathogenic | Biallelic *NTHL1* case |
| 23 | Pat_005 | CCFR | 61 | F | pMMR | 1x adenoma, 1x CRC | *NTHL1* | c.244C>T p.(Gln82Ter) \| c.211dup p.(Ala71GlyfsTer2) | Compound heterozygous | (Likely) pathogenic \| (Likely) pathogenic | Biallelic *NTHL1* case |
| 24 | Pat_110 | CCFR | 61 | F | pMMR | 1x CRC | *NTHL1* | c.835C>T p.(Gln279Ter) \| c.244C>T p.(Gln82Ter) | Heterozygous | (Likely) pathogenic | Monoallelic *NTHL1* case |
| 25 | Pat_108 | CCFR | 43 | M | pMMR | 1x CRC | *NTHL1* | c.244C>T p.(Gln82Ter) | Heterozygous | (Likely) pathogenic | Monoallelic *NTHL1* case |

*Abbreviations: ID, identification number; Pat, patient; Rel, relative; CCFR, Colon Cancer Family Registry; ANGELS, Applying Novel Genomic approaches to Early-onset and suspected Lynch Syndrome colorectal and endometrial cancers; GCPS, The Genetics of Colonic Polyposis Study; CRC, colorectal cancer; MMR, DNA mismatch repair; pMMR, DNA mismatch repair proficient; dMMR, DNA mismatch repair deficient.*
