# Supplementary figures and images for: Adenomas from individuals with pathogenic biallelic variants in the MUTYH and NTHL1 genes demonstrate base excision repair tumour mutational signature profiles similar to colorectal cancers, expanding potential diagnostic and variant classification applications
Source: Transl Oncol. 2025 Jan 9;52:102266. doi: 10.1016/j.tranon.2024.102266 (PMC11774829; doi:10.1016/j.tranon.2024.102266)

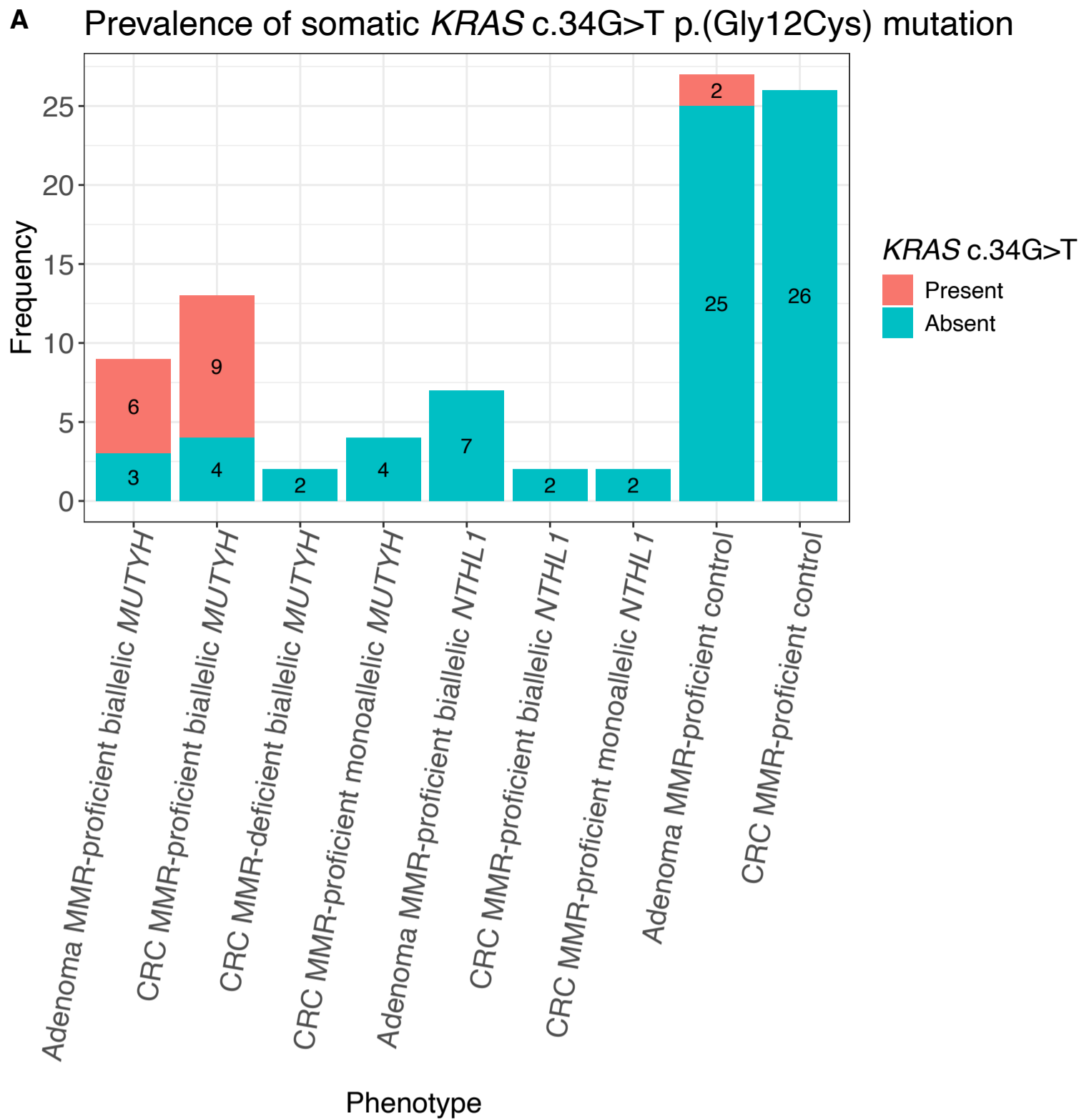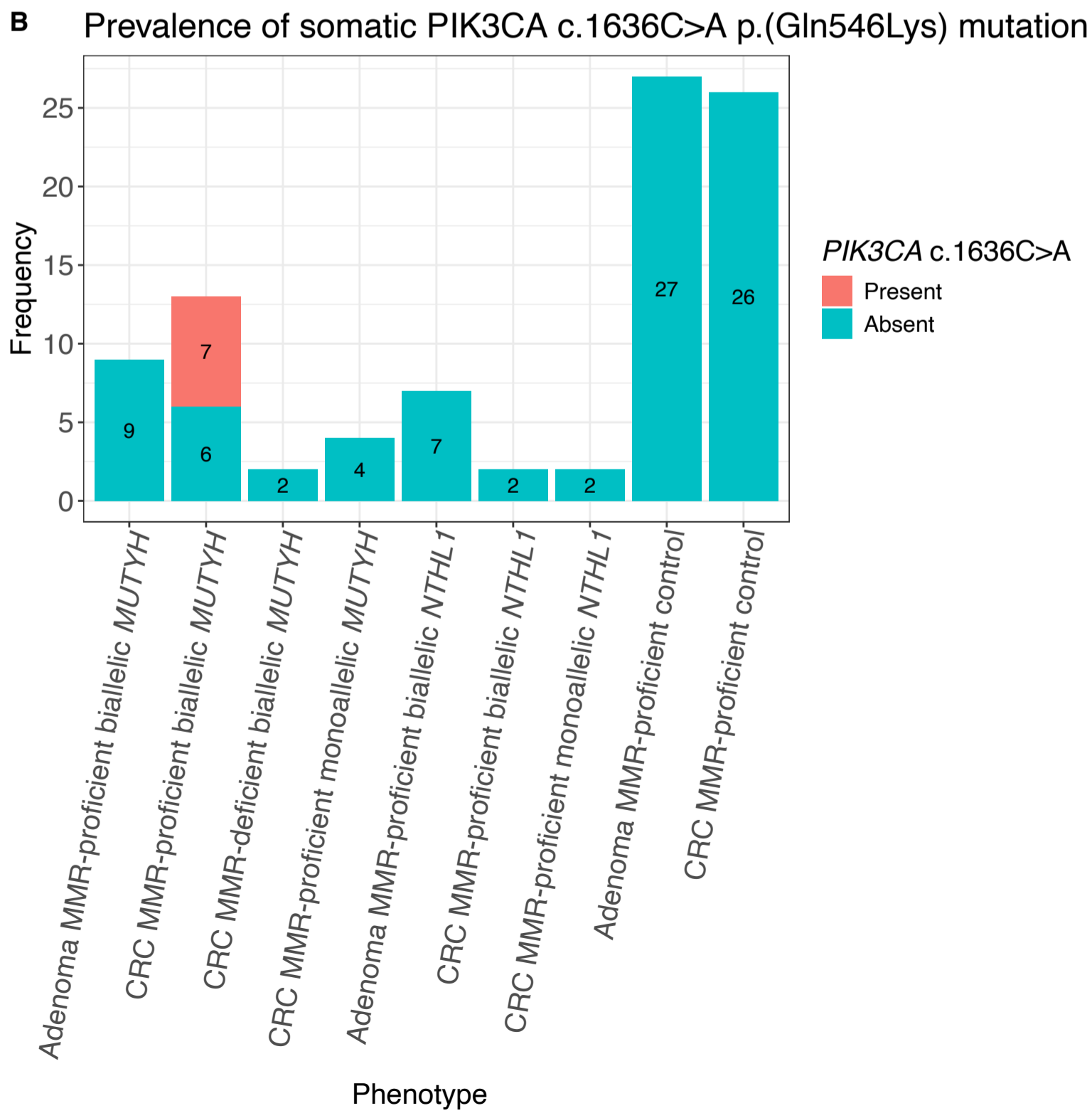

Supplement: Supplementary file 2 [file mmc2.pdf]
